# Rice field snail shell anticancer properties: An exploration opinion

**DOI:** 10.3389/fonc.2022.1078981

**Published:** 2023-01-13

**Authors:** Joice Junita Imelda Rompas, Sylvia Laatung, William Ben Gunawan, Iftitan Setya Widayanti, Vincentius Mario Yusuf, Timotius William Yusuf, Netty Salindeho, Mrinal Samtiya, Fahrul Nurkolis

**Affiliations:** ^1^ Animal Science Study Programme, Faculty of Animal Husbandry, Sam Ratulangi University, Manado, Indonesia; ^2^ Nutrition Science Department, Faculty of Medicine, Diponegoro University, Semarang, Indonesia; ^3^ Medical Programme, Faculty of Medicine, Universitas Brawijaya, Malang, Indonesia; ^4^ Dentistry Programme, Faculty of Dentistry, Trisakti University, Jakarta, Indonesia; ^5^ Fishery Products Technology Study Program, Faculty of Fisheries and Marine Sciences, Sam Ratulangi University, Manado, Indonesia; ^6^ Department of Nutrition Biology, Central University of Haryana, Mahendragarh, India; ^7^ Biological Sciences, State Islamic University of Sunan Kalijaga (UIN Sunan Kalijaga), Yogyakarta, Indonesia

**Keywords:** anticancer, *Pila ampullacea*, rice snail shell, nutraceuticals, molecular and cellular oncology, cancer

## 1 Introduction

Mollusks, especially Gastropods – which include land, freshwater, and sea snails – are commonly used as traditional medicine and cost-effective food resource (1). Snail meat has beneficial nutritional values as it is high in protein and low in fat (2). Hence markets around Asia, such as Indonesia, China, Taiwan, Japan, and Hongkong, frequently process snail meat into food (3). A type of freshwater snail*, Pila ampullacea*, is a native mollusk easily found in Southeast Asian rice fields and lakes. Like its fellow gastropods, it contains high nutritional values (100 mg meat: ± 209 kcal calories, ± 18 g protein, 12 mg zinc, 102 mg iron, and 812 mg calcium) (4). Although conventionally consumed as food, *Pila ampullacea* is also recognized as a crop pest (5). The only component of snails that has been incorporated into food is snail meat (6, 7). As their consumption rises, snail shells are less explored functionally and become animal feed material, accessories, and waste products with low economic value (1).

Calcium carbonate constitutes 87-96% of the total weight of freshwater snail shells (8). High dietary calcium intake is clinically protective against multiple chronic diseases, including lowering the risks of developing cancer (9, 10). Calcium carbonate is also the primary material used to synthesize hydroxyapatite, a biocompatible material with high binding activity to proteins and genetic materials. Nanoparticle hydroxyapatite showed *in vitro* and *in vivo* anti-proliferative potential against cancer cells (11, 12). Various studies have shown that snail shells also contain bioactive compounds such as chitin – the primary chitosan material – that offer antipathogenic, antioxidant properties, as well as pharmaceutical additive potential (13, 14). Furthermore, chitin and its derivatives were found to have a significant immunomodulating response against cancer and antitumor activity through the downregulation of tumor angiogenesis factors, apoptotic effects stimulation, and decreased cell adhesion (15). However, the bioactive component’s profile of snail shells is partially influenced by their habitat, surrounding environment, mineral content, and microorganisms (1). Therefore, this article specifically aims to summarize the recent findings on potential anticancer properties in molecular and cellular oncology mechanisms of rice field snail shells.

## 2 Rice snail in general

Freshwater snails or rice field snails (*Pila ampullacea*) belong to a genus of large aquatic snails and the family of *Ampullariidae* (16). Besides, rice field snails can generally be found in ponds, marshes, and lakes. It has a morphology similar to a golden snail (*Pomacea canaliculata*) but with a darker green to black spiral-shaped shell. Its approximate height is around 100 mm, with a width of up to 100 mm (5). *Pila ampullacea* – also named apple snail – is often regarded as a crop pest with potential damage of 10-40% to wetland agricultural goods, especially rice (17). *Pila ampullacea* feeds on aquatic plants such as lettuce; however, during its starvation phase, it can consume decaying animals (18). Rice snail is considered to be a potential functional food ingredient due to its high protein and calcium with low fat and phosphorus content, which can offer metabolic advantages such as augmenting weight loss and reducing cardiometabolic risks (19, 20). *Pila ampullacea* has also been processed into various types of food and formulations, such as baby porridge, liquid food formula, crackers, and flavor enhancers (5, 16, 17). Furthermore, high scavenging activity towards free radicals was found in snail extract, potentially due to its amino acid characteristics (21).

## 3 Anticancer properties of rice snail shell

The shell extract of snails has been studied for its antioxidant property and influence on the Caco-2 cancer cell line (22). Interestingly, even though the antioxidant activity of the shell extract was higher than other parts of the snail, the shell extract didn’t display a significant reduction in cancer cell line viability, which may be influenced by the presence of Fe. Further study also showed the snail shell caused significant inhibitory effects against several cancer cell lines (SKOV-3, MCF-7, MDA-MB-231, and HepG2), in which the antiproliferative effect against SKO-V-3 (human ovarian cancer) cells was comparable to cisplatin as its positive control (23). Surprisingly, snail shells – in the form of powder – exhibited wound-healing properties (24), reflecting the anti-inflammatory activity of the shells. This regulation of the inflammatory process may be beneficial in treating cancer since inflammation and wound healing share “similar” mechanisms and hallmarks to cancer (25).

Hydroxyapatite (Ca_10_(PO_4_)_6_(OH)_2_) was also successfully synthesized from the rice field snail shells (26). Hydroxyapatite – which is highly contained in snail shells – exhibited anticancer properties possibly through endocytosis in cancer cells and cellular protein synthesis suppression (27, 28). Direct injection of hydroxyapatite nanoparticles into a transplanted tumor formed by human hepatocarcinoma cells *in vivo* showed a 50% reduction in tumor size and inhibited the proliferation of cancer cells >65% (28). Hydroxyapatite nanoparticles exert their effects by localizing around the endoplasmic reticulum of the cancer cells where they impede the translation process by competitively binding to the ribosome and preventing mRNA from bonding with it; which causes GO/G1 phase arrest in the cell cycle (28). Another similar study showed laminated hydroxyapatite (L-HAp) significantly decreased the migration ability of human breast cancer MDA-MB-231 cells by blocking integrin β-1 phosphorylation which mediates the adhesion of cancer cells (29).

Proximate analysis of many shells of snail species revealed that snail shells contain low iron and zinc but high calcium and magnesium (30). Calcium and magnesium intakes were associated with the incidence of cancers and patients’ survivability (31, 32). Snail shell also contains calcium carbonate (87-96% of shell weight) (8). Interventions using calcium carbonate showed the capability of calcium carbonate to prevent recurrent adenomas in colorectal cancer patients (33). On the other side, calcium carbonate has been studied as a targeted drug or gene delivery strategy for malignant tissues and cells (34) and as a compartment of the cancer imaging system (35).

Apart from the aforementioned properties of snail shells, snail shell’s functionality as metal adsorbents due to their chitin, chitosan, and hydroxyapatite content has also been studied (36–38), indicating their potential to prevent cancer caused by carcinogenic metal toxicity (39). Chitin has an antiproliferative effect by lowering cell viability which can be employed in cancer treatment as a carrier for delivering medicines to a specific spot (40). Moreover, chitosan and its derivatives are also known for their anti-inflammatory, antioxidant, and anticancer properties (41). On the other hand, Matusiewicz et al. (22) also identified myristic acid in the shell extract, a medium-chain fatty acid that can cause cancer cell death (42).

## 4 Future applications and implications

Snail meat is the only part of snails that has been utilized in various food products to increase their protein and calorie content which can alleviate malnutrition (6, 11). The snail shells were mostly underutilized and ended up as waste. Snail shell powder has only been used as a calcium source in the diets of broilers, small animals, and cattle (43). However, snail shell remains interesting to be utilized as food, supplement, or drug component. Incorporating snail shell powder into a diet may be a novel form of mineral fortification. For example, a snail shell can be made into a powder that can be added to daily food or beverages. Dietary intake of chitosan – which is present in the snail shells – may promote cellular immunity, which is strongly linked to cancer development (44). Chitosan was capable to boost antigen-specific T helper 1 response in a type I interferon receptor-dependent manner with high tolerability and immunoreactivity (44). Snail shells can be innovated into a skeletal health supplement since snail shells are rich in calcium and magnesium (30) which maintain the integrity of the bone (45). Looking ahead, the rich calcium carbonate in snail shells may also be incorporated with other compounds, such as tocopheryl polyethylene glycol succinate and curcumin (46) to create a more potent drug that can overcome cancer drug resistance and reverse tumor immunosuppression. Interestingly, Huang et al. (35) also mentioned the usage of calcium carbonate nanoparticles in cancer imaging, further highlighting the potential of rice snail shells to be applied as a calcium carbonate source since it has good bioavailability (87-96%).

## 5 Discussions

**Figure 1:**
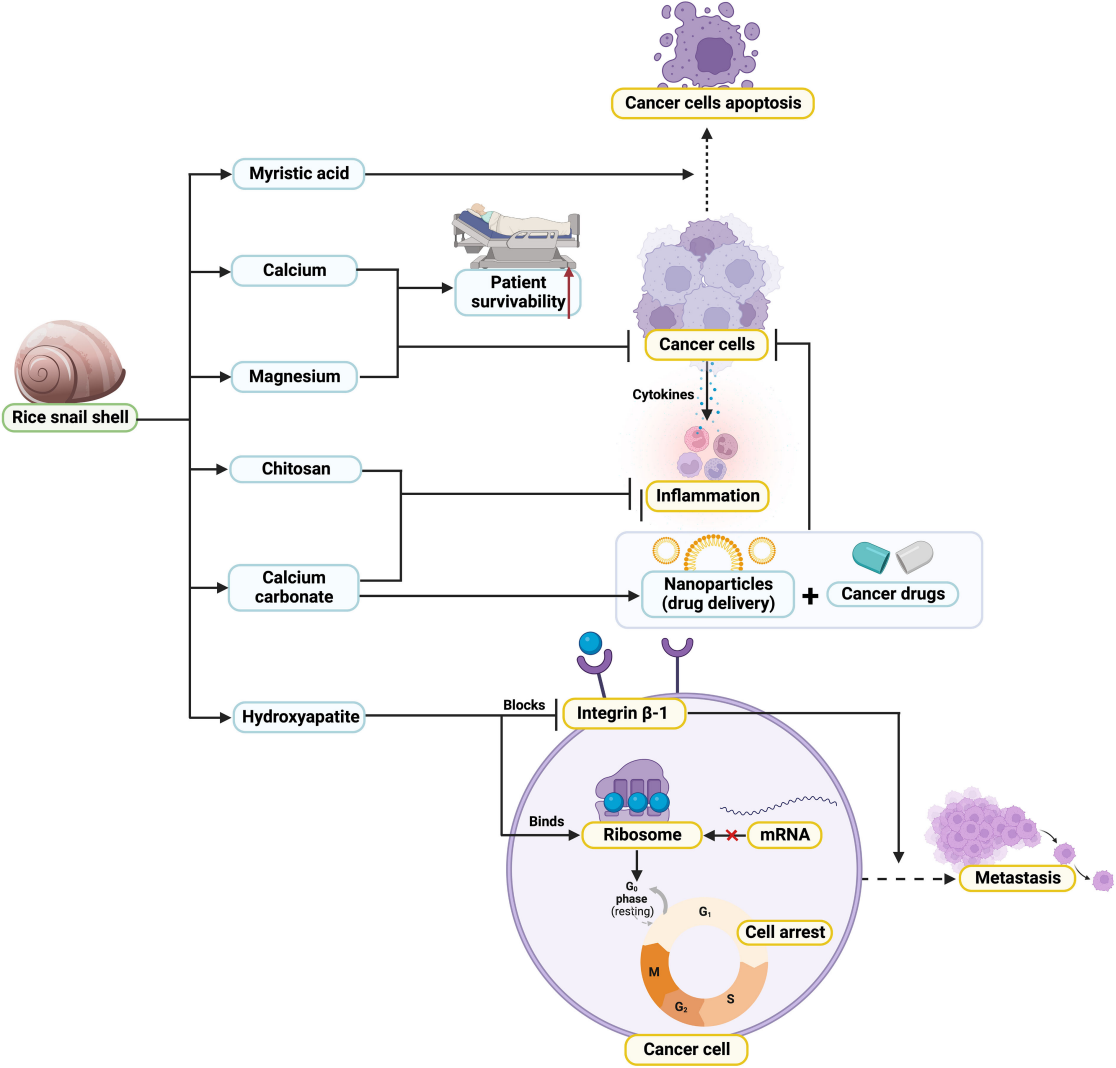
Possible mechanistic of anticancer properties of rice snail shell.

According to previous reports, it has been summarized that snail shells are a rich source of calcium and magnesium. Calcium is a vital element that is needed by the body to function and is only taken by the body through dietary sources (**Figure 1**). Both calcium and magnesium also play a vital role in the mineralization of the skeleton and possess a broad range of functions (47) such as anti-cancer activity. Previous mice study also suggested that dietary intake rich in calcium could help to reduce colon cancer (48). A recent meta-analysis study indicated that higher dietary calcium intake could lower esophageal cancer risks (9). The latest study found that snails contain CaCO_3_ crystals in their shell with diverse shell surface functional groups (**Figure 1**) (8). Calcium carbonate has been investigated as a cancer imaging system compartment and as a tailored medicine or gene delivery technique for malignant tissues and cells (34, 35). CaCO_3_ is also one of the essential components for the synthesis of hydroxyapatite where hydroxyapatite nanoparticles are used for cancer treatment (**Figure 1**). Hydroxyapatite inhibited cancer mainly through the translation and phosphorylation processes of cancer cells (28, 29). The presence of myristic acid in the rice snail shells exhibited therapeutic potential for cancer by inhibiting the inflammation/autophagy pathways in cancer cells (42). On the other hand, the biological properties of chitin and chitosan may contribute to the diagnosis and therapy of cancer (49).

Overall, this opinion proposes that snail shells have promising potential to help reduce cancer concerns (**Figure 1**). However, this is only an exploratory opinion at the moment, so large *in vivo* and *in vitro* trials will be needed to conclude that snail shells have anti-cancerous properties.

## Author contributions

JR, SL, WG, IW, VY, TY, NS, MS, and FN: Contributed to the conceptualization with the design of the critical opinion study, firstly drafted the manuscript, edited-revised, and approved the final version of the submitted manuscript. All authors contributed to the article and approved the submitted version.

## References

[1] Sundalian M Husein SG Putri NKD. Review: Analysis and benefit of shells content of freshwater and land snails from gastropods class. Biointerface Res Appl Chem (2021) 12:508–17. doi: 10.33263/BRIAC121.508517

[2] Ulagesan S Kim H. Antibacterial and antifungal activities of proteins extracted from seven different snails. Appl Sci (2018) 8:1362–71. doi: 10.3390/app8081362

[3] Needham S Funge-Smith SJ. The consumption of fish and fish products in the Asia-Pacific region based on household surveys. FAO Regional Office for Asia and the Pacific, Bangkok, Thailand: RAP Publication (2014). p. 87. Available at: https://www.fao.org/apfic/publications/detail/en/c/396958/.

[4] Nurhasan M Maehre HK Malde MK Stormo SK Halwart M James D. Nutritional composition of aquatic species in Laotian rice field ecosystems. J Food Composit Anal (2010) 23(3):205–13. doi: 10.1016/j.jfca.2009.12.001

[5] Broto RTDW Arifan F Setyati WA Eldiarosa K Zein AR. Crackers from fresh water snail (Pila ampullacea) waste as alternative nutritious food. IOP Conf Ser: Earth Environ Sci (2020) 448448:1–4. doi: 10.1088/1755-1315/448/1/012039

[6] Adeyeye SAO Bolaji OT Abegunde TA Adesina TO. Processing and utilization of snail meat in alleviating protein malnutrition in Africa: A review. Nutr Food Sci (2020) 50(6):1085–97. doi: 10.1108/NFS-08-2019-0261

[7] Ghosh S Jung C Meyer-Rochow VB. Snail as mini-livestock: Nutritional potential of farmed *Pomacea canaliculata* (*Ampullariidae*). Agric Natural Resour (2017) 51:504–11. doi: 10.1016/j.anres.2017.12.007

[8] Parveen S Chakraborty A Chanda DK Pramanik S Barik A Aditya G. Microstructure analysis and chemical and mechanical characterization of the shells of three freshwater snails. ACS Omega (2020) 5(40):25757–71. doi: 10.1021/acsomega.0c03064 PMC7557267 33073101

[9] Li Q Cui L Tian Y Cui H Li L Dou W. Protective effect of dietary calcium intake on esophageal cancer risk: a meta-analysis of observational studies. Nutrients (2017) 9(5):510. doi: 10.3390/nu9050510 28524093 PMC5452240

[10] Peterlik M Grant WB Cross HS. Calcium, vitamin d and cancer. Anticancer Res (2009) 29(9):3687–98. 19667166

[11] Kargozar S Mollazadeh S Kermani F Webster TJ Nazarnezhad S Hamzehlou S. Hydroxyapatite nanoparticles for improved cancer theranostics. J Funct Biomat (2022) 13(3):100. doi: 10.3390/jfb13030100 PMC9326646 35893468

[12] Zhao H Wu C Gao D Chen S Zhu Y Sun J. Antitumor effect by hydroxyapatite nanospheres: Activation of mitochondria-dependent apoptosis and negative regulation of phosphatidylinositol-3-kinase/protein kinase b pathway. ACS nano (2018) 12(8):7838–54. doi: 10.1021/acsnano.8b01996 30059628

[13] Jatto OE Asia IO Medjor WE. Proximate and mineral composition of different species of snail shell. Pac J Sci Technol (2010) 11(1):416–9.

[14] Abd El-Hack ME El-Saadony MT Shafi ME Zabermawi NM Arif M Batiha GE. Antimicrobial and antioxidant properties of chitosan and its derivatives and their applications: A review. Int J Biol Macromol (2020) 164:2726–44. doi: 10.1016/j.ijbiomac.2020.08.153 32841671

[15] Satitsri S Muanprasat C. Chitin and chitosan derivatives as biomaterial resources for biological and biomedical applications. Molecules (2020) 25(24):5961. doi: 10.3390/molecules25245961 33339290 PMC7766609

[16] Ihsani I Diana Nur Afifah DNA Anantyo A Mulyono M Teddy Wahyu Nugroho T Firdaus Wahyudi F. Analysis of nutrient content and shelf life from freshwater snail (Pila ampullacea) instant baby porridge. Food Res (2020) 4(Suppl. 3):184–196. doi: 10.26656/fr.2017.4(S3).S20

[17] Fatimah I Aulia GR Puspitasari W Nurillahi R Sopia L Herianto R. Microwave-synthesized hydroxyapatite from paddy field snail (Pila ampullacea) shell for adsorption of bichromate ion. Sustain Environ Res (2018) 28(6):462–71. doi: 10.1016/j.serj.2018.10.003

[18] Lamkom T Phosri D. Study on gonadosomatic index of Thai native apple snail (Pila ampullacea linneaus 1758) in the rice fields of srimuang-mai district, ubon ratchathani and effect of diet on the growth of juveniles. J Fish Environ (2017) 41(1):27–36.

[19] Widiany FL Sja’bani M Huriyati E. The organoleptic quality of liquid food formula made from snail (*Pila ampullacea*), tempeh, and moringa leaves. Slovak J Food Sci (2021) 15 :961–9. doi: 10.5219/1672

[20] Wycherley TP Moran LJ Clifton PM Noakes M Brinkworth GD. Effects of energy-restricted high-protein, low-fat compared with standard-protein, low-fat diets: A meta-analysis of randomized controlled trials. Am J Clin Nutr (2012) 96(6):1281–98. doi: 10.3945/ajcn.112.044321 23097268

[21] Ulagesan S Kuppusamy A Kim HJ. Antimicrobial and antioxidant activities of protein hydrolysate from terrestrial snail *Cryptozona bistrialis* . J Appl Pharm Sci (2018) 8:12–9. doi: 10.7324/JAPS.2018.81202

[22] Matusiewicz M Kosieradzka I Niemiec T Grodzik M Antushevich H Strojny B. *In vitro* influence of extracts from snail helix aspersa müller on the colon cancer cell line caco-2. Int J Mol Sci (2018) 19(4):1064. doi: 10.3390/ijms19041064 29614018 PMC5979351

[23] Alburae NA Mohammed AE. Antiproliferative effect of the red sea cone snail, *Conus geographus* . Trop J Pharm Res (2020) 19(3):pp.577–581. doi: 10.4314/tjpr.v19i3.17

[24] Andrade PHM Portugal LC Rondon ES Kadri MCT Matos MFC. Effect of powdered shells treatment of the snail *Megalobulimus lopesi* on wounds of diabetic rats. Acta Cir Bras (2018) 33(2):185–96. doi: 10.1590/s0102-865020180020000010 29513817

[25] MacCarthy-Morrogh L Martin P. The hallmarks of cancer are also the hallmarks of wound healing. Sci Signal (2020) 13(648):eaay8690. doi: 10.1126/scisignal.aay8690 32900881

[26] Charlena Suparto I Putri D. Synthesis of hydroxyapatite from rice fields snail shell (*Bellamya javanica*) through wet method and pore modification using chitosan. Proc Chem (2015) 17:27–35. doi: 10.1016/j.proche.2015.12.120

[27] Tang W Yuan Y Liu C Wu Y Lu X Qian J. Differential cytotoxicity and particle action of hydroxyapatite nanoparticles in human cancer cells. Nanomedicine (2014) 9(3):397–412. doi: 10.2217/nnm.12.217 23614636

[28] Han Y Li S Cao X Yuan L Wang Y Yin Y. Different inhibitory effect and mechanism of hydroxyapatite nanoparticles on normal cells and cancer cells in vitro and *in vivo* . Sci Rep (2014) 4:7134. doi: 10.1038/srep07134 25409543 PMC4238015

[29] Jin J Zuo G Xiong G Luo H Li Q Ma C. The inhibition of lamellar hydroxyapatite and lamellar magnetic hydroxyapatite on the migration and adhesion of breast cancer cells. J Mat Sci: Mat Med (2014) 25(4):1025–31. doi: 10.1007/s10856-013-5126-8 24363068

[30] Nkansah MA Agyei EA Opoku F. Mineral and proximate composition of the meat and shell of three snail species. Heliyon (2021) 7(10):e08149. doi: 10.1016/j.heliyon.2021.e08149 34746458 PMC8551499

[31] Gong TT Wei YF Li X Liu FH Wen ZY Yan S. Pre-diagnostic dietary consumption of calcium and magnesium and calcium-to-magnesium intake ratio and ovarian cancer mortality: results from the ovarian cancer follow-up study (OOPS). Eur J Nutr (2022) 61:3487–97. doi: 10.1007/s00394-022-02883-2 35596007

[32] Shah SC Dai Q Zhu X Peek RM Jr Roumie C Shrubsole MJ. Associations between calcium and magnesium intake and the risk of incident oesophageal cancer: an analysis of the NIH-AARP diet and health study prospective cohort. Br J Cancer (2020) 122(12):1857–64. doi: 10.1038/s41416-020-0818-6 PMC7283350 32242097

[33] Chu DZ Hussey MA Alberts DS Meyskens FL Jr Fenoglio-Preiser CM Rivkin SE. Colorectal chemoprevention pilot study (SWOG-9041), randomized and placebo controlled: the importance of multiple luminal lesions. Clin Colorectal Cancer (2011) 10(4):310–6. doi: 10.1016/j.clcc.2011.06.005 PMC4286321 21782524

[34] Dizaj SM Sharifi S Ahmadian E Eftekhari A Adibkia K Lotfipour F. An update on calcium carbonate nanoparticles as cancer drug/gene delivery system. Expert Opin Drug Deliv (2019) 16(4):331–45. doi: 10.1080/17425247.2019.1587408 30807242

[35] Huang H Zhang W Liu Z Guo H Zhang P. Smart responsive-calcium carbonate nanoparticles for dual-model cancer imaging and treatment. Ultrasonics (2020) 108:106198. doi: 10.1016/j.ultras.2020.106198 32590261

[36] Asimeng BO Amenyaglo EK Dodoo-Arhin D Efavi JK Kwakye-Awuah B Tiburu EK. Snail based carbonated-hydroxyapatite material as adsorbents for water iron (II). Mat (Basel) (2022) 15(9):3253. doi: 10.3390/ma15093253 PMC9104755 35591586

[37] Bambaeero A Bazargan-Lari R. Simultaneous removal of copper and zinc ions by low cost natural snail shell/hydroxyapatite/chitosan composite. Chin J Chem Eng (2021) 33:221–30. doi: 10.1016/j.cjche.2020.07.066

[38] Foroutan R Oujifard A Papari F Esmaeili H. Calcined *Umbonium vestiarium* snail shell as an efficient adsorbent for treatment of wastewater containing Co (II). 3 Biotech (2019) 9(3):78. doi: 10.1007/s13205-019-1575-1 PMC6372419 30800589

[39] Kim HS Kim YJ Seo YR. An overview of carcinogenic heavy metal: Molecular toxicity mechanism and prevention. J Cancer Prev (2015) 20(4):232–40. doi: 10.15430/JCP.2015.20.4.232 PMC4699750 26734585

[40] Baharlouei P Rahman A. Chitin and chitosan: Prospective biomedical applications in drug delivery, cancer treatment, and wound healing. Mar Drugs (2022) 20(7):460. doi: 10.3390/md20070460 35877753 PMC9319611

[41] Kim S. Competitive biological activities of chitosan and its derivatives: Antimicrobial, antioxidant, anticancer, and anti-inflammatory activities. Int J Polymer Sci (2018) 2018: 1708172. doi: 10.1155/2018/1708172

[42] Park S Kim M Hong Y Lee H Tran Q Kim C. Myristoylated TMEM39AS41, a cell-permeable peptide, causes lung cancer cell death. Toxicol Res (2020) 36(2):123–30. doi: 10.1007/s43188-020-00038-1 PMC7099121 32257924

[43] Tchakounte F Kana JR Azine P Meffowoet CP Djuidje VP. Effects of dietary level of calcium on body proportion and nutritional value of African giant snail (*Archachatina marginata*). Anim Res Vete Sci (2019) 3. doi: 10.24966/ARVS-3751/100020

[44] Carroll EC Jin L Mori A Muñoz-Wolf N Oleszycka E Moran HBT. The vaccine adjuvant chitosan promotes cellular immunity *via* DNA sensor cGAS-STING-Dependent induction of type I interferons. Immunity (2016) 44(3):597–608. doi: 10.1016/j.immuni.2016.02.004 26944200 PMC4852885

[45] Capozzi A Scambia G Lello S. Calcium, vitamin d, vitamin K2, and magnesium supplementation and skeletal health. Maturitas (2020) 140:55–63. doi: 10.1016/j.maturitas.2020.05.020 32972636

[46] Guan YY Zeng SQ Qin Y Mu Y Liu H. Vitamin e-tocopheryl polyethylene glycol succinate decorated drug delivery system with synergistic antitumor effects to reverse drug resistance and immunosuppression. J Colsurfa (2021) 628:127387. doi: 10.1016/j.colsurfa.2021.127387

[47] Peacock M. Calcium metabolism in health and disease. Clin J Am Soc Nephrol (2010) 5(Supplement 1):S23–30. doi: 10.2215/CJN.05910809 20089499

[48] Yang K Lamprecht SA Shinozaki H Fan K Yang W Newmark HL. Dietary calcium and cholecalciferol modulate cyclin D1 expression, apoptosis, and tumorigenesis in intestine of adenomatous polyposis coli1638N/+ mice. J Nutr (2008) 138(9):1658–63. doi: 10.1093/jn/138.9.1658 18716166

[49] Karagozlu MZ Kim SK. Anticancer effects of chitin and chitosan derivatives. Adv Food Nutr Res (2014) 72:215–25. doi: 10.1016/B978-0-12-800269-8.00012-9 25081085

